# Comparison of Leukocyte-Rich and Leukocyte-Poor Platelet-Rich Plasma on Pressure Ulcer in a Rat Model

**DOI:** 10.1093/jbcr/irac191

**Published:** 2023-01-02

**Authors:** Ze Yuan, Yanxue Wang, Yudan Li, Caina Lin, Shaoling Wang, Junchao Wang, Chao Ma, Shaoling Wu

**Affiliations:** Department of Rehabilitation Medicine, Sun Yat-sen Memorial Hospital, Sun Yat-sen University, Guangzhou, Guangdong, China; Department of Rehabilitation Medicine, Sun Yat-sen Memorial Hospital, Sun Yat-sen University, Guangzhou, Guangdong, China; Department of Ultrasound, The Third People’s Hospital of Shenzhen, Shenzhen, Guangdong, China; Department of Rehabilitation Medicine, Sun Yat-sen Memorial Hospital, Sun Yat-sen University, Guangzhou, Guangdong, China; Department of Rehabilitation Medicine, Sun Yat-sen Memorial Hospital, Sun Yat-sen University, Guangzhou, Guangdong, China; Department of Rehabilitation Medicine, Sun Yat-sen Memorial Hospital, Sun Yat-sen University, Guangzhou, Guangdong, China; Department of Rehabilitation Medicine, Sun Yat-sen Memorial Hospital, Sun Yat-sen University, Guangzhou, Guangdong, China; Department of Rehabilitation Medicine, Sun Yat-sen Memorial Hospital, Sun Yat-sen University, Guangzhou, Guangdong, China

## Abstract

Pressure ulcer (PU) is a common type of chronic wound that is difficult to treat. Platelet-rich plasma (PRP) is rich in cytokines and growth factors, and it can be divided into two categories according to its leukocyte content: leukocyte-poor PRP (P-PRP) and leukocyte-rich PRP (L-PRP). PRP has been applied in a variety of wound treatments, due to its strong ability to promote repair. This study aims to investigate the therapeutic effects of PRP on PU and elucidate the role of leukocytes in the treatment process. Sprague-Dawley rats were used to establish PU models of ischemia–reperfusion injury by applying magnets externally. L-PRP, P-PRP, and saline were injected into the dermal wounds. Wound healing analysis and sampling were performed on days 3, 7, 11, and 15 after treatment. Histological examinations, real-time PCR, immunohistochemical examinations, and biomechanical assay were carried out on the wound samples. The PRP groups exhibited greater wound inflammatory response than the control group in the early stage but the response reduced rapidly as the wound healed. On days 7, 11, and 15, the PRP groups also yielded better wound healing rates and histological outcomes than the control group, with superior biomechanical properties observed on day 15. Among both PRP groups, the L-PRP group attained a higher wound healing rate than the P-PRP group on day 7, with greater significant early inflammatory responses, and more prominent angiogenesis. Therefore, PRP is proven to accelerate the healing of PU, with L-PRP being more effective in regulating inflammation and promoting angiogenesis than P-PRP.

Pressure ulcer (PU) is a common type of chronic wound that usually develops on skin that covers bony apophysis, whereby the skin and/or underlying tissues are often damaged due to local skin pressure, and it is often hard to treat.^1^ PU mainly affects elderly people, patients with spinal cord injuries, patients who require special care requirements, or patients who have undergone undergoing orthopedic surgery. With the number of long-term bedridden patients on the rise due to the aging society, PU cases have become more and more common. Not only does it seriously impact patients’ health and quality of life, PU also exerts a heavy financial burden on the healthcare system.^2^ Common treatments for PU include surgical removal of necrotic tissue, negative-pressure wound therapy, application of appropriate dressings, moist wound healing, improvement of nutrition metabolism, posture management, and more.^3^ However, these treatments do not produce reliable and satisfactory results in many cases. As a result, there is growing interest in developing new advanced therapies to address problematic wounds like PU.

Platelet-rich plasma (PRP) is obtained from centrifugation of the blood, with a concentration of platelets at three to five times the physiological concentration. PRP is rich in fibrin and various growth factors, which are involved in mediating cell growth, cell proliferation, and chemotaxis, besides promoting the synthesis of extracellular matrix and playing a key role in tissue repair.^4^ Studies have shown that the concentration of leukocytes would affect the concentration of cell cytokines and growth factors in PRP.^5^ Therefore, PRP can be divided into leukocyte-rich PRP (L-PRP) and leukocyte-poor PRP (P-PRP) according to its concentration of leukocytes.^6^ Due to the safety of its source and the low cost of preparation, PRP possesses a broad market prospect and clinical value.

Clinical trials have reported that the therapeutic effects of PRP on chronic wounds, such as diabetic foot ulcers, lower-extremity venous ulcers, and burns.^7–9^ L-PRP also exhibited positive effect on few PU trails.^10,11^ Nevertheless, some studies indicated that leukocytes may impose detrimental effects on tissue healing instead due to their role in the release of inflammatory cytokines and increased matrix metalloproteinases (MMP) levels.^12–14^ As there was no head-to-head study between L-PRP and P-PRP, no evidence is currently available to compare the effectiveness of both on PU. Hence, this current study focuses on studying the therapeutic effects and mechanism of PRP on PU, and the relationship of leukocytes concentration on this therapeutic effect by treating PU rats with L-PRP and P-PRP, in the hopes of discovering more evidence and guidance for the use of PRP in treating PU clinically.

## MATERIALS AND METHODS

### Animals

A total of 60 male Sprague-Dawley rats were used in this experiment. The said rats were fed adaptively in a single cage for 2 to 3 weeks until reaching 6 to 8 weeks of age and weighed 260 to 300 g. The whole experiment was carried out in a pathogen-free environment. Among them, 8 rats were used for the preparation of PRP, 4 rats were categorized as the normal group, 16 rats as the control group, and the remaining 32 rats as the experimental groups.

### PRP Preparation

**Figure 1.**
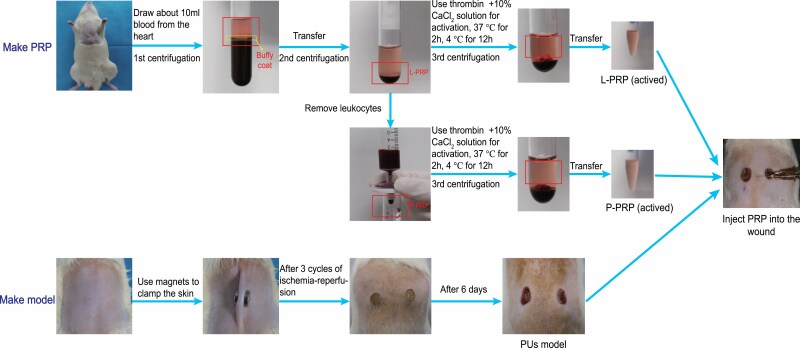
The processes involved in PRP preparation and PU models establishment. *PRP*, platelet-rich plasma; *PU*, pressure ulcer.

The process involved in PRP preparation is shown in Figure 1. Eight rats were anesthetized with 2% sodium pentobarbital solution (2 ml/kg). Whole blood was subsequently collected from the heart of these rats and anticoagulated with sodium citrate. Centrifugation is the key step in PRP preparation. The first round of centrifugation was carried out with the following conditions: 500*g*, 10 minutes, 4°C; the supernatant was then aspirated with a disposable pipette to 1 to 2 mm below the buffy coat. The specimen was again centrifuged but with the following conditions: 1000*g*, 10 minutes, 4°C; half of the supernatant was removed and shaken to concentrate the platelets. L-PRP was obtained after two centrifugations, and half of this L-PRP was filtered through a leukocyte filter (Sterile Acrodisc WBC Syringe Filter, Pall) to obtain P-PRP. The leukocyte concentration and number of platelet in P-PRP, L-PRP, and whole blood were then measured by the Mindray BC-5000Vet analyzer, respectively. A mixture of 100 U/ml thrombin (Solarbio) and 10% CaCl_2_ solution was added to PRP at a ratio of 1:9 for activation. The mixture was then kept at 37°C for 2 hours, and 4°C for 12 hours. Finally, the activated PRP was collected after the third round of centrifugation at 3000*g*, 20 minutes, and 4°C; the activated PRP was stored at −80°C until being used.

### Wound Model and Treatment Protocol

The noninvasive method reported by Istvan was used to make the PU model in this experiment.^15,16^ As shown in Figure 1, a 7 cm × 5 cm area was selected on the lower back of the rats. After the hair was removed from this selected area exposing local skin removal, two circular permanent magnets (Tongyong Magnet, China) with 2700 G magnetic force at 4 mm in thickness and 12 mm in diameter were placed. The skin was clamped by these two magnets for 12 hours to achieve ischemia, and then removed for the next 12 hours to allow reperfusion. After three cycles of ischemia–reperfusion, the wound was left to develop naturally for another 6 days until PU appeared. The activity of these rats was not limited throughout this whole process.

A total of 48 rat models were successfully established and were divided into 3 groups at random. These groups were treated with L-PRP, P-PRP, and saline solution, respectively. After anesthesia, 100 µl of L-PRP, P-PRP, or saline solution was injected into the base of the wound edge and center with a 4.5 UI needle. The local complexion of the skin was observed after injection. Only one injection/treatment was given per rat.

### Wound Healing Analysis

After treatment, the wounds were photographed with a camera on days 0, 3, 7, 11, and 15. The software ImageJ version 1.53c was used to measure wound area and calculate healing rates. The healing rate was calculated with the following formula:


$$\begin{array}{ll} Healing\;rate=[1-(Wound\;area\;D_{n})/\\ & (Wound\;\;\;area\;D_{0})]\times 100\% \end{array}$$

### Histological Examinations

Sampling was performed on days 3, 7, 11, and 15 after treatment. At each time point, four rats from each group were sacrificed. Wounds and 5 mm normal skin tissue around were harvested from the left back of experimental rats, and cut into two parts subsequently. Half of the samples were then fixed with 4% paraformaldehyde and then embedded in paraffin. Sections at a thickness of 5 µm were used for hematoxylin–eosin (H&E) staining, Masson staining, and subsequent immunohistochemical staining. A semiquantitative scoring system was used in a blinded manner to assess re-epithelization, and the presence of polymorphonuclear leukocytes (indicating inflammation) upon H&E staining (score 0: absent; 1: minimal; 2: mild; 3: moderate; and 4: marked).^17^ Whereas, the ratio of collagen in the skin sample was calculated by the image analysis software ImageJ version 1.53c upon Masson staining.

### Real-Time Quantitative PCR

Another half of the samples mentioned above were cut into pieces, and ground with small steel balls in a cold mill. Total RNA was isolated from the samples by using Trizol (Invitrogen) according to the manufacturer’s instructions. Reverse transcription from total RNA (1000 ng) was performed using Evo M-MLV RT Mix Kit (AG11728, Accurate Biotechnology, Hunan, Co., Ltd) with gDNA Clean for qPCR. RT-PCR reactions were carried out using the SYBR Green PCR Kit (AG11701, Accurate Biotechnology, Hunan, Co., Ltd). After a 30-s predegeneration at 95°C, 40 cycles were performed: denaturation at 95 seconds for 15 seconds, extension at 60°C for 30 seconds followed by a 65 to 95°C solubility curve, which was constructed to analyze the fluorescence signal. The relative amount of mRNA was normalized against GAPDH (Supplementary Information 1). The primers (Table 1) were produced by Shanghai Sangong Biotechnology (Shanghai, China). The relative expression levels were determined by the 2^−ΔΔCT^ method and calculated relative to the control group.

**Table 1.** RT-qPCR primer sequences

No.	Primer	Sequence (5ʹ–3ʹ)
1	IL-6-FORWARD	AGTTGCCTTCTTGGGACTGATGTTG
2	IL-6-REVERSE	GGTATCCTCTGTGAAGTCTCCTCTCC
3	TNF-α-FORWARD	AAAGGACACCATGAGCACGGAAAG
4	TNF-α-REVERSE	CGCCACGAGCAGGAATGAGAAG
5	IL-10-FORWARD	GGCAGTGGAGCAGGTGAAGAATG
6	IL-10-REVERSE	TGTCACGTAGGCTTCTATGCAGTTG
7	MMP9-FORWARD	CACCGCCAACTATGACCAGGATAAG
8	MMP9-REVERSE	CTGCTTGCCCAGGAAGACGAAG
9	VEGF-FORWARD	CGGTGTGGTCTTTCGTCCTTCTTAG
10	VEGF-REVERSE	AGGGATGGGTTTGTCGTGTTTCTG

### Immunohistochemical Examinations

Dewaxed skin sections from samples were incubated with 3% H_2_O_2_ for 10 minutes. After sealing with 3% BSA, these sections were incubated with the following primary antibody of IL-6, IL-1β, IFN-γ, TGF-β1, α-SMA, and MMP-9 (Supplementary Information 2); and then stained with secondary antibody. Next, these sections were washed with PBS buffer solution for three times. The 3,3ʹ-diaminobenzidine (DAB) reagent was used to develop the color and hematoxylin was used as a counterstain. Finally, the sections were viewed and analyzed under the microscope (XSP-C204, CIC). The nuclei that were stained by hematoxylin appeared blue, while the positive expression of DAB manifested as brown. The ImageJ software version 1.53c was used to measure the mean density of the positive expression area.

### Biomechanical Assay

The wound on the right back of each rat was used for biomechanical testing. During sampling, the sore and surrounding normal skin tissue were excised (9 mm × 25 mm), soaked in saline solution, and stored at −30°C until tested. The biomechanical assay was carried out at room temperature. The skin tissue was cut into three strips at sizes of 25 mm (length) × 3mm (width) and measured by a vernier caliper to ensure the thickness. The two ends were clamped on the jig of the tensile testing machine (ZQ990A, China) and stretched at a speed of 10 mm/min until samples were broken. The stress–strain curve was drawn for relevant results: 1) The maximum force—the force bore by the leather strip during the process of stretching leading up to breaking; 2) Tensile strength—dividing the maximum force over the cross-sectional area, characterizing the resistance of the material to maximum uniform plastic deformation; 3) The maximum force elongation—the ratio of deformation degree to the original length when the leather strip was stretched at maximum force.

### Statistical Analysis

Statistical data were analyzed by GraphPad and SPSS 26.0 version 9. Quantitative group data with a homogeneous variance and normal distribution were compared using one-way ANOVA; whereas qualitative group data were compared by chi-square test or rank-sum test (Mann–Whitney *U*). Test level *α* = 0.05, *P* values < .05 were considered as being statistically significant.

## RESULTS

### PRP Test Results

In whole blood, the number of leukocytes was 5.0 ± 0.6 (10^6^ cells/ml) while the number of platelets was 5.9 ± 1.1 (10^8^ cells/ml). In L-PRP, the number of leukocytes was 7.7 ± 3.5 (10^6^ cells/ml) whereas the number of platelets was 3.2 ± 0.17 (10^9^ cells/ml). In P-PRP, the number of leukocytes was 3.0 ± 0.7 (10^4^ cells/ml), while the number of platelets was 2.8 ± 0.14 (10^9^ cells/ml).

### Wound Healing Rate

**Figure 2.**
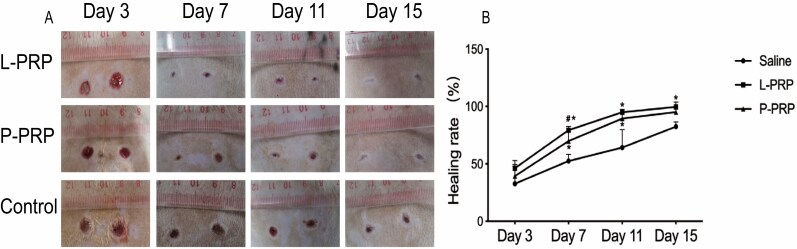
The measurement of wound areas. A. Photographs of wounds in the three groups at 3, 7, 11, and 15 days after treatment. B. Relative wound healing rate in different groups at 3, 7, 11, and 15 days after treatment. * represents *P* < .05 for PRP groups compared to the control group; # represents *P* < .05 for the L-PRP group compared to the P-PRP group. *L-PRP*, leukocyte-rich PRP; *P-PRP*, leukocyte-poor PRP; *PRP*, platelet-rich plasma.

The result of wound healing analysis is shown in Figure 2. During the entire experiment, the wound healing rate of rats in the PRP groups was higher than the control group. The differences were significant on days 7, 11, and 15 in the L-PRP group (*P* < .05), while it was only significant on days 7 and 11 in the P-PRP group (*P* < .05). The healing rate of the L-PRP group was higher than the P-PRP group at each time point, but the differences were not significant (*P* > .05) except for day 7 (*P* < .05).

### Histological Analysis

**Figure 3.**
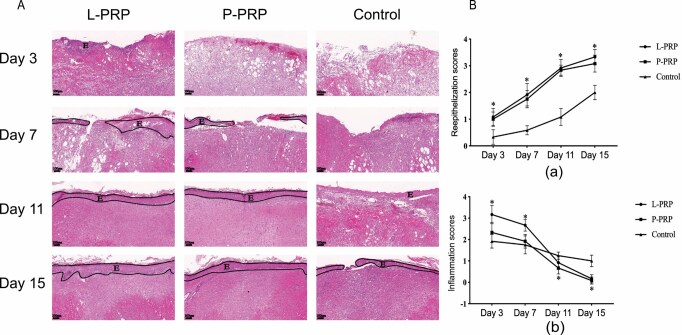
The analyses of histological change on wounds. A. Samples were subjected to H&E staining. Representative images on the central surface of skin tissues in the wound are shown. E represents the epidermis layer. B. Relative semiquantitative scores of the three groups at four different time points. Re-epithelialization scores is shown in picture (a), and inflammation scores in (b). * represents *P* < .05 for PRP groups compared to the control group. *PRP*, platelet-rich plasma.

**Figure 4.**
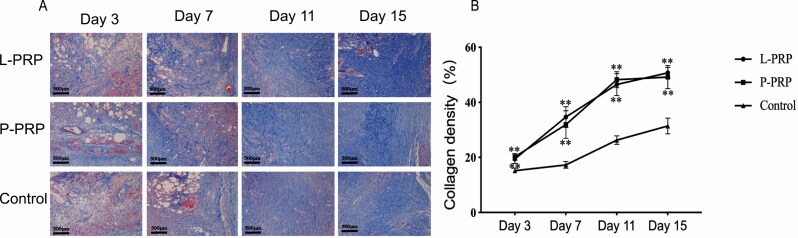
The analyses of collagen synthesis on wounds. A. Samples were subjected to Masson staining. Central portions of skin wounds are shown. B. The ratio of collagen in skin tissue from the wound is shown. ** represents *P* < .01 for PRP groups compared to the control group. *PRP*, platelet-rich plasma.

The outcome of H&E staining is shown in Figure 3A. As tissue repair progresses, inflammation gradually decreases, blood vessels gradually proliferate, collagen synthesis gradually increases, and the wound site was gradually covered by epithelium. The semiquantitative histological score is shown in Figure 3B. The re-epithelization scores increased with time, and were higher in the L-PRP group (*P* < .05) and P-PRP group (*P* > .05) at each time point as compared to the control group, but no difference was observed among the PRP groups (*P* > .05). The degree of inflammation in the L-PRP group was higher than the control group on days 3 and 7 (*P* < .05), but lower than the control group after 11 days (*P* > .05); meanwhile, the degree of inflammation in the P-PRP group was higher than the control group on days 3 and 7 (*P* > .05), but lower than the control group after 11 days (*P* < .05). The result of Masson staining is shown in Figure 4. The collagen scores of both PRP groups were higher than that of the control group at all time points (*P* < .05), but no difference was observed among the PRP groups (*P* > .05).

### Gene Relative Expressions

**Figure 5.**
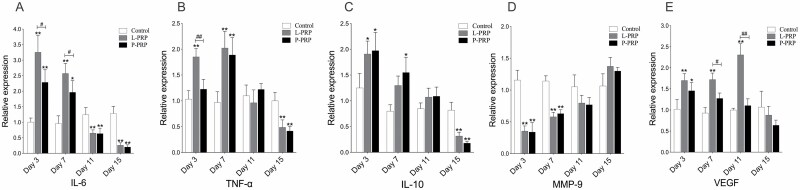
mRNA expression of IL-6 (A), TNF-α (B), IL-10 (C), MMP-9 (D), and VEGF (E) in skin wounds. *(or #) represents *P* < .05 whereas **(or ##) represents *P* < .01. * represents PRP groups compared to the control group while # represents the L-PRP group compared to the P-PRP group. *L-PRP*, leukocyte-rich PRP; *P-PRP*, leukocyte-poor PRP; *PRP*, platelet-rich plasma.

Real-time quantitative PCR was used to detect the gene expression of IL-6, TNF-α, IL-10, MMP-9, and VEGF in skin tissue from the wound. As shown in Figure 5, IL-6 expressions in both PRP groups were significantly higher than the control group (*P* < .05) on days 3 and 7, with the L-PRP group yielding higher expression than the P-PRP group (*P* < .05). On the contrary, the levels of IL-6 in both PRP groups were significantly lower than the control group (*P* < .05) on days 11 and 15. Meanwhile, TNF-α expression was higher in the L-PRP group than both the P-PRP and control groups (*P* < .05) on day 3, but both PRP groups exhibited higher expression than the control group on day 7 (*P* < .05) before lowering on day 15 (*P* < .05). The expressions of IL-10 in both PRP groups were higher than the control group on day 3 (*P* < .05), lower on day 15 (*P* < .05), and the P-PRP group was higher than the control group on day 7 (*P* < .05). The MMP-9 levels of both PRP groups were lower than the control group on days 3 and 7 (*P* < .05), with an increasing trend over time. On day 3, the VEGF levels in both PRP groups were higher than the control group (*P* < .05) on day 3, and the L-PRP group was higher than the P-PRP and control groups on days 7 and 11 (*P* < .05).

### Immunohistochemical Analysis

#### IL-6, IL-1β, and IFN-γ

**Figure 6.**
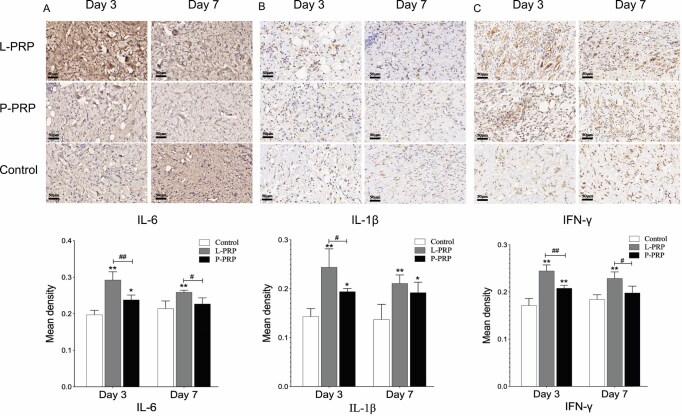
The proteins expression of relevant inflammatory factors. Pictures A, B, and C show tissue sections that were subjected to immunostaining with IL-6, IL-1β, and IFN-γ, respectively. Representative images from the wounds are shown. The ratio of the positive areas in these samples is shown below. *(or #) represents *P* < .05 whereas **(or ##) represents *P* < .01. * represents PRP groups compared to the control group while # represents the L-PRP group compared to the P-PRP group. *L-PRP*, leukocyte-rich PRP; *P-PRP*, leukocyte-poor PRP; *PRP*, platelet-rich plasma.

As shown in Figure 6, the IL-6, IL-1β, and IFN-γ protein levels of both PRP groups were significantly higher than the control group (*P* < .05), with the L-PRP group showing higher levels than the P-PRP group on day 3 (*P* < .05). On day 7, the protein levels of IL-6 and IFN-γ were higher in the L-PRP group than the P-PRP and control groups (*P* < .05), whereas the IL-1β protein level was higher in both PRP groups when compared to the control group (*P* < .05). As healing progresses, the inflammatory cytokines in both PRP groups gradually decreased (Supplementary Figure S2).

#### TGF-β1, α-SMA, and MMP-9

**Figure 7.**
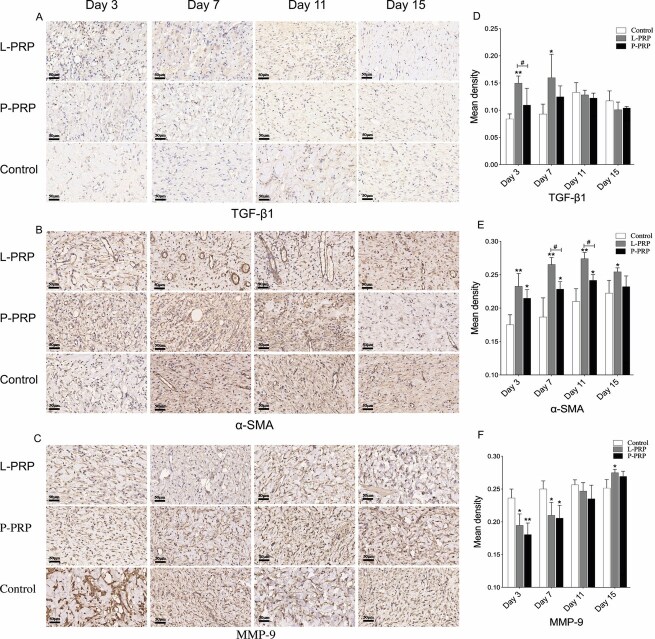
The expression of proteins for regulating extracellular matrix and angiogenesis. Pictures A, B, and C show tissue sections that were subjected to immunostaining with TGF-β1, α-SMA, and MMP-9, respectively. Representative images from the wounds are shown. The mean density of the positive areas in these samples is shown in D–F. *(or #) represents *P* < .05 whereas ** represents *P* < .01. * represents PRP groups compared to the control group while # represents the L-PRP group compared to the P-PRP group. *L-PRP*, leukocyte-rich PRP; *P-PRP*, leukocyte-poor PRP; *PRP*, platelet-rich plasma.

As shown in Figure 7, the level of TGF-β1 protein was higher in the L-PRP group than the control group on days 3 and 7 (*P* < .05), and higher than the P-PRP group on day 3 (*P* < .05). The expression of the α-SMA protein reflects the number of functional arterioles and myofibroblasts. The α-SMA protein level was also higher in both PRP groups compared to the control group on days 3, 7, and 11 (*P* < .05), whereas the α-SMA protein level in the L-PRP group was higher than the P-PRP group, especially on days 7 and 11 (*P* < .05). Throughout the whole experiment, the expression of TGF-β1 and α-SMA proteins in both PRP groups showed an increasing trend before reversing at a later stage. On days 3 and 7, the protein expression of MMP-9 in both PRP groups was lower than the control group (*P* < .05). As tissue healing entered the remodeling stage, the MMP-9 protein expression in both PRP groups gradually increased. On day 15, the expression of MMP-9 protein was higher in the L-PRP group as compared to the control group (*P* < .05). The expression of MMP-9 protein in the L-PPR group was slightly higher than the P-PPR group at every time points (*P* > .05).

### Biomechanical Assay

**Figure 8.**
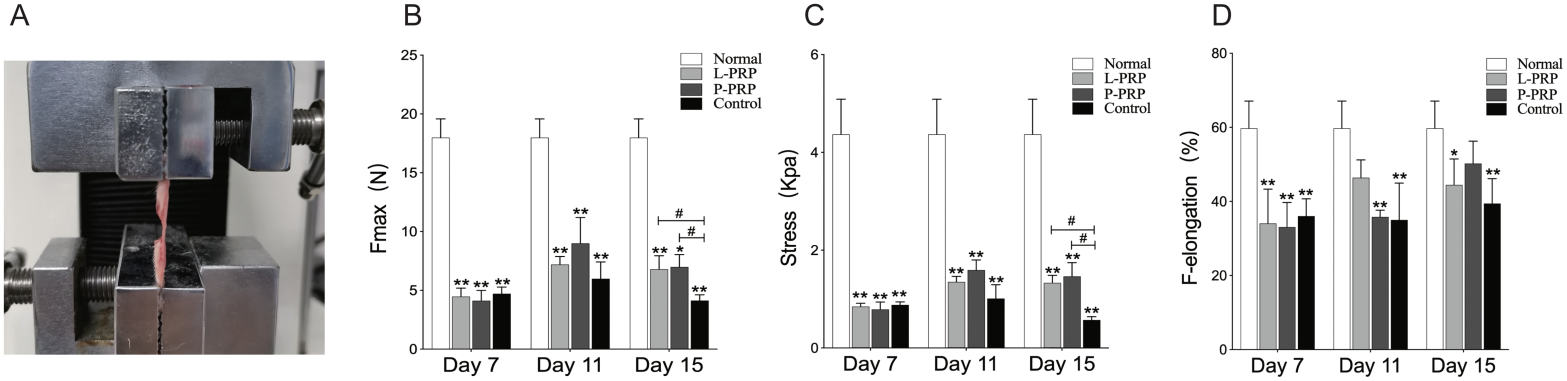
The biomechanical testing of wound skins. A. Photograph of a biomechanical assay in testing. The two ends were clamped on the jig of the tensile testing machine. Comparing maximum force (B), stress (C), and maximum force elongation (D) among the normal, PRP, and control groups during the healing period. *(or #) represents *P* < .05 whereas ** represents *P* < .01. * represents PRP and control groups compared to the normal group while # represents PRP groups compared to the control group. *PRP*, platelet-rich plasma.

As shown in Figure 8, the biomechanical properties of the skin samples from PRP and control groups were worse than those from the normal group (*P* < .05). No difference was observed in the maximum force and strength between the PRP groups and the control group on days 7 and 11. Only on day 15, the skin stress and maximum force of PRP groups were higher than the control group (*P* < .05). On the 11th day, the maximum force elongation in the L-PRP group was approximative to that of the normal group, whereas the P-PRP group achieved a similar approximation on the 15th day.

## DISCUSSION

Results from this experiment show the ability of L-PRP and P-PRP in promoting the healing of PU, with better histological performance and biomechanical results.

### Inflammation

The application of PRP significantly enhanced the inflammatory responses in the wound at the early stage (especially in the L-PRP group), but the inflammatory response was gradually reduced as the wound healing progressed. According to the relative research, PRP increases macrophage infiltration and upregulates the expression level of inflammatory factors, which ultimately contribute to wound healing, and these effects are related to the platelet and leukocyte content in PRP.^18,19^ In this study, the L-PRP group expressed more inflammatory mediators (IL-6, TNF-α, IL-1β, and IFN-γ) than the other groups. It was pointed out that a large number of erythrocytes and granulocytes in L-PRP secrete many inflammatory mediators, while the lymphocytes in P-PRP mainly secrete anti-inflammatory mediators.^20^ Similar studies have shown that the leukocytes in PRP release high levels of TNF-α and IL-6, leading to the promotion of inflammation and matrix degradation, which are detrimental to tissue healing.^21,22^ Nevertheless, the leukocytes in PRP are also shown to be beneficial in stimulating an anti-infection and immune response besides promoting cell chemotaxis, proliferation, and differentiation.^23^ Besides, circulating inflammatory cells (neutrophils, macrophages, and lymphocytes) could be attracted and recruited to the injury site by chemotaxis. The recruited cells then release another wave of pro-inflammatory cytokines (such as TNF-α and interleukins) and growth factors (FGF and VEGF), which are helpful in stimulating keratinocytes, fibroblasts, and angiogenesis to promote tissue regeneration, thus driving the transition to the proliferative phase of healing.^24,25^

### Blood Vessel

Results of PCR and immunohistochemical analysis showed that PRP increased the secretion of VEGF and promote vascular proliferation during the tissue repair stage, indicating the possible role of PRP in increasing blood supply to injured tissues to promote wound healing. Early recovery of blood flow would raise relative cells from bone marrow and peripheral blood to induce tissue repair.^26^ In the later stage, blood vessels were remodeled faster in the PRP groups. Similar study had also found that the application of gel + PRP could induce the synthesis of endogenous VEGF and increase the area of capillaries in the wound.^27^ A study had proven that VEGF and PDGF are both derived from leukocytes and platelets.^28^ Our study found that the angiogenesis was more obvious in the L-PRP group than the P-PRP group, with a higher level of VEGF and α-SMA in the process of tissue repair. Additionally, the increase in TGF-β1 release and activation in the L-PRP group may also promote the expression of α-SMA protein.^29^ Consequently, the blood supply was more abundant in the L-PRP group, which is crucial to accelerate tissue repair at the early stage.

### Extracellular Matrix

At early stage of tissue repair, the collagen bundles were thin and discontinuous with lots of fibroblasts, and they would gradually grow denser and became more orderly arranged at later stage.^30^ Upon staining of skin tissue in the PRP groups, collagen showed the tendency of a dense, wavy parallel arrangement during the later stage. This proved that the synthesis of collagen could be accelerated with the use of PRP (especially L-PRP). One possible explanation to this might be the direct stimulation of local stem cells by the TGF-β1 contained in PRP to accelerate the synthesis of collagen fibers and promote the transformation of fibroblasts into myofibroblasts, both of which are crucial in the secretion of the extracellular matrix.^31,32^ In addition, inflammatory response causes the release of cytokines and growth factors, which induce the chemotaxis of fibroblasts and angiogenesis, thus stimulating the synthesis of collagen.^33,34^ MMP-9 promotes the migration of keratinocytes and participates in the degradation or remodeling of the extracellular matrix.^35^ Henceforth, the overproduction or high activity level of MMP-9 leads to poor wound healing in skin wounds caused by chronic ulcer.^32^ PCR and immunohistochemistry results pointed out that the expression of MMP-9 in the PRP groups was low at the early stage and gradually increased later. This is similar to the result of Farghali, who used PRF to treat skin wounds in canines,^36^ and found that PPR might reduce the tissue matrix degradation in the early stage to promote tissue repair, and increases the expression of MMP-9 at a later stage to promote reorganization and remodeling. Another study also found that wounds treated with PRP showed faster collagen maturation and epithelial differentiation, which might be attributed to the increase of MMP-9.^37^

### Epithelium

H&E staining showed that the PRP groups achieved wound epithelialization earlier and shortened the time taken for epithelial remodeling, but little difference was noticed between them. From appearance to healing, a wound will undergo the processes of inflammation, cell proliferation, and remodeling. The proliferation or migration of keratinocytes is a key step in accelerating wound healing, which are often impaired in skin ulcer, and consequently lead to the slow formation of epithelial cells.^38,39^ In our experiment, the application of L-PRP promoted the TGF-β1 expression at the early stage of wound healing, which could induce the migration of keratinocytes and promote re-epithelization.^40^ Besides, a study similar to ours had shown that PRP treatment could induce the proliferation and migration of HaCaT keratinocytes, with 90% to 100% epithelialization observed on an average of 15.18 days after treatment.

### Limitation

Firstly, healthy rats have strong self-healing ability which may affect their ability to heal. Secondly, this study did not observe the stage when the wound was completely repaired into normal tissue, which might have an impact on fully evaluating the therapeutic effect of PRP treatment in the biomechanical aspect.

## CONCLUSION

**Figure 9.**
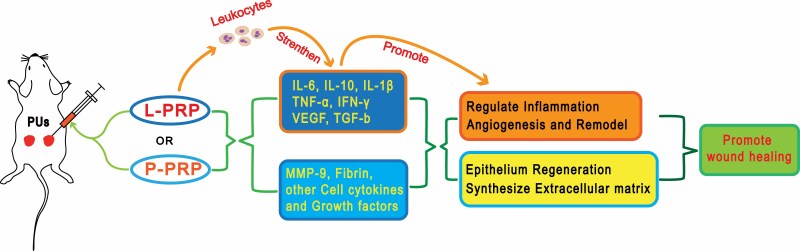
The potential mechanism of L-PRP and P-PRP on treating PU. *L-PRP*, leukocyte-rich PRP; *P-PRP*, leukocyte-poor PRP; *PU*, pressure ulcer.

Results from this study evidently show the potential of PRP in accelerating PU healing with L-PRP inducing tissues to express more inflammatory factors and growth factors that are related to wound healing. The therapeutic mechanism of local PRP injection may include: regulating the inflammatory response of the wound, promoting the proliferation of skin cells, improving anabolism, increasing the formation of blood vessels, and enhancing biomechanical properties (Figure 9). This study may provide some guidelines for the clinical application of L-PRP on treating PU.

## Supplementary Material

irac191_suppl_Supplementary_Material

## Notes

*Funding*: The authors gratefully acknowledge financial support from the Sun Yat-Sen Clinical Research Cultivating Program (SYS-C-202002), Sun Yat-sen Medical-Industrial Integration Project (YXYGRH202201), and Project funded by Shenzhen Third People’s Hospital (No. G2021005).

*Ethical approval*: This experiment had been approved by the Animal Ethics Committee of Sun Yat-sen University (SYSU-IACUC-2022-000119), and the rats involved were treated in accordance with the relevant regulations.

*Authors’ contributions*: Ze Yuan: conception, design, operation, analysis, and drafting; Yanxue Wang: conception, design, operation, assessment, and revision; Yudan Li: conception, assessment, and revision; Caina Lin: design, analysis, and revision; Shaoling Wang: design and revision; Junchao Wang: analysis and revision; Chao Ma: organization, conception, revision, and corresponding; Shaoling Wu: organization, conception, analysis, drafting, and corresponding.
