# Supplementary material for: Comparison of Leukocyte-Rich and Leukocyte-Poor Platelet-Rich Plasma on Pressure Ulcer in a Rat Model
Source: J Burn Care Res. 2023 Jan 2;44(4):860–8. doi: 10.1093/jbcr/irac191 (PMC10321391; doi:10.1093/jbcr/irac191)
Supplement: irac191_suppl_Supplementary_Material [file irac191_suppl_supplementary_material.docx]

# Supplementary Information

Supplementary Information for *“Comparison of Leukocyte-Rich and Leukocyte-Poor Platelet-Rich Plasma on Pressure ulcer in a Rat Model”*

1. **qRT-PCR assays**

***(1). First Strand cDNA Synthesis***

The cDNA was synthesized with10 μl reaction system. The reaction mixture was gently mixed, centrifuged briefly, and then incubated for 15 minutes at 37°C. The reaction was terminated by heating it at 85°C for 5 seconds.

Table S1. Reaction mixture:

| **Component** | **Volume** |
| --- | --- |
| 5 x Evo M-MLV RT Master Mix（AG11706） | 2 μl |
| Total RNA | 1000 ng |
| RNase free water | Add to 20 μl |

***(2). Preparation of PCR Master Mix***

Table S2. For each 10µL reaction, prepare the following reaction mix:

| **Component** | **Volume** |
| --- | --- |
| 2 x SYBR® Green Pro Taq HS Premix (AG11701) | 5 μl |
| F/R Primers | 0.2 μl /0.2 μl |
| cDNA | 1 μl |
| RNase free water | 3.6 μl |

Table S3. PCR amplification:

| **Stage 1** | **Stage 2（40 cycles）** | **Stage 3（Melt Curve）** |
| --- | --- | --- |
| 95℃, 30s  Pre-denaturation | 95℃, 15s   Denaturation  60℃, 30s   Annealing/Extension | 65℃→95℃ |

1. **Immunohistochemical examinations**

Table S4. Information of primary antibodies used in this study

| Primary antibody | Application | Company | Catalog Number | RRID |
| --- | --- | --- | --- | --- |
| IL-6 | 1: 200 | Affinity | DF6087 | AB_2838055 |
| IL-1β | 1: 800 | Servicebio | GB11113 | / |
| IFN-γ | 1: 400 | Affinity | DF6045 | [AB_2838015](https://antibodyregistry.org/search.php?q=AB_2838015) |
| TGF-β1 | 1: 800 | BOSTER | BA0290 | / |
| α-SMA | 1: 200 | Affinity | AF1032 | AB_2835329 |
| MMP-9 | 1: 200 | Affinity | AF5228 | AB_2837714 |

**3.**


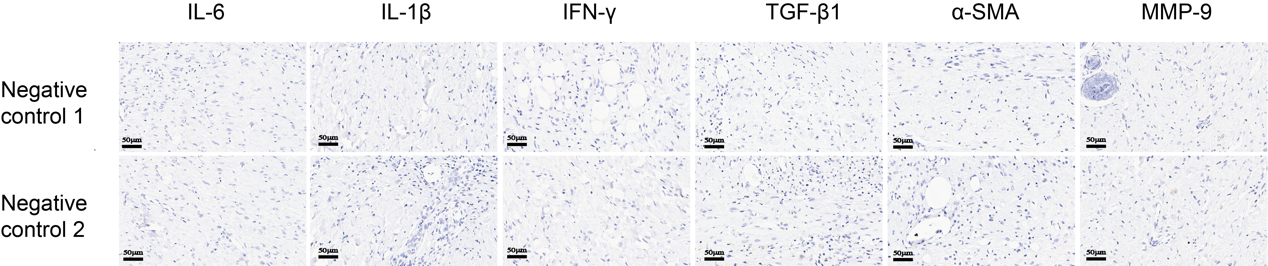


**Figure S1.** The negative control staining of immunohistochemical examinations.

4.


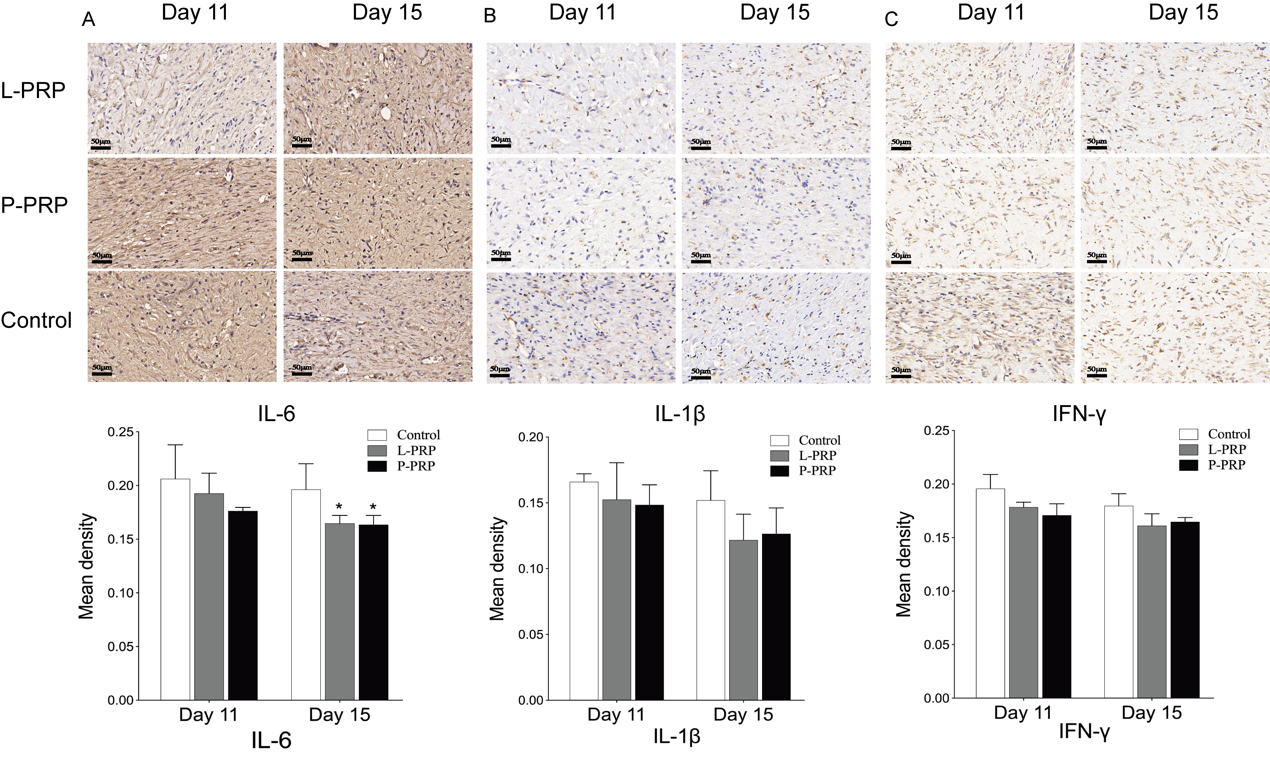


**Figure S2.** The proteins expression of relevant inflammatory factors. Pictures A, B and C show tissue sections that were subjected to immunostaining with IL-6, IL-1β and IFN-γ respectively. Representative images from the wound were shown. The mean density of the positive area in samples is shown below. * represents *P* < 0.05, for PRP groups compared to the control group.
